# The clinical effectiveness and complications of lumbar selective fenestration and concave-side fusion (LSFCF) in degenerative lumbar scoliosis (DLS) combined with lumbar spinal stenosis (LSS)

**DOI:** 10.1186/s12893-022-01842-2

**Published:** 2022-11-22

**Authors:** Yang Hou, Hongyang Shi, Haoyang Shi, Tianyi Zhao, Jiangang Shi, Guodong Shi

**Affiliations:** Department of Orthopaedic Surgery, Changzheng Hospital, Second Military Medical University, No. 415 Fengyang Rd, Shanghai, 200003 China

**Keywords:** LSFCF, DLS, LSS, VAS, ODI

## Abstract

**Purpose:**

This retrospective study was performed to analyze the clinical effects and complications of LSFCF in the surgical treatment of DLS combined with lumbar spinal stenosis (LSS).

**Methods:**

A total of 26 eligible patients (mean age, 64.73 y; 17 men, 9 women) with DLS combined with LSS were included and LSFCF surgery was performed. An independent spine surgeon retrospectively reviewed the medical records and radiographs of all patients to evaluate surgical data and surgery-related complications. Preoperative, postoperative, and follow-up questionnaires were obtained to assess clinical outcomes.

**Results:**

The average follow-up period of this study was 20.14 ± 5.21 months. The operation time and blood loss of patients underwent LSFCF were 129.33 ± 15.74 min and 356.13 ± 21.28 ml. The clinical effects of all patients in terms of visual analogue scale (VAS) and Oswestry disability index (ODI) have been significantly improved at the final follow-up postoperatively (*P* < 0.05). Complications such as infection, cerebrospinal fluid leakage, nerve injury, and internal fixation failure, etc. were not observed during the follow-up period.

**Conclusion:**

The LSFCF surgery is a safe and effective treatment for DLS patients combined with LSS.

## Background

Degenerative lumbar scoliosis (DLS) is adult scoliosis, which is a deformity caused by degenerative changes of the spine in patients with mature bones. DLS is mainly seen in patients over 50 years of age, with a cobb angle of more than 10°, and the incidence of DLS is about 6% [1, 2]. The occurrence of DLS is related to the degenerative changes of the physiological structure of the lumbar spine, including intervertebral discs, facet joints and ligaments, etc. [3, 4]. The imaging examination of DLS mainly shows the coronal deformity caused by vertebral rotation. In addition, the asymmetrical wedging of the disc due to degeneration could lead to rotary and lateral subluxation, which further increases the degree of coronal deformity [5, 6]. DLS is mainly manifested as recurrent low back pain, which is caused by facet joint arthrosis, disc degeneration, and the loss of lumbar lordosis [7]. Patients may present with symptoms of radicular pain in the lower extremities due to nerve compression mainly on the concave side (nerve passage narrowing) or on the convex side (nerves being pulled and displaced) [6, 8, 9]. In addition, if lumbar spinal stenosis is combined, the patient can also show symptoms of neurogenic claudication [10].

Surgical treatment is an effective method for DLS patients with no significant effect after conservative treatment. The posterior fusion with pedicle screw instrumentation in addition to decompression of neural elements has been widely used in the surgical treatment of DLS, which can effectively inhibit the deterioration of scoliosis and improve the sagittal imbalance [10–12]. Previous studies have confirmed that short segment fixation and fusion can achieve good clinical results in the surgical treatment of patients with DLS, and its complications are significantly reduced compared with long segment fixation [10, 13]. In the current study, we adopted a novel short segment fusion method, named as lumbar selective fenestration and concave-side fusion (LSFCF) in surgical treatment of patients with DLS in combination with lumbar spinal stenosis (LSS). The objective of this study is to investigate the clinical effects and complications of LSFCF for cases with DLS and LSS, and provide the reliable basis for surgical management.

## Methods

### Study design

This is a retrospective study.

### Study population

This study has been approved by the ethics committee of Shanghai Changzheng Hospital. We retrospectively reviewed the data of patients with DLS who underwent surgical treatment in our hospital from January 2018 to March 2020.

The inclusion criteria

(1) All patients had different degrees of chronic low back pain and neurological symptoms of lower extremities (neurogenic claudication or sensory or/and motor symptoms or cauda equina syndrome), and multi-segmental LSS was demonstrated by MRI; (2) Presence of adult scoliosis, defined by a coronal Cobb angle above 10°; (3) all patients had no significant improvement after conservative treatment for at least 6 months; (4) patients aged 50 years and over.

The exclusion criteria

(1) History of lumbar spinal surgery; (2) other spinal diseases (e.g., ankylosing spondylitis, spine tumor, fracture, or neurologic disorders); (3) unwillingness to complete study questionnaires; (4) non-adherence to clinical and radiographic follow-up protocols.

### Patient demographics

A total of 26 eligible patients (mean age, 64.73 y; 17 men, 9 women) were enrolled in the current study based on data of our department. In the current study, patients underwent LSFCF included 4 cases with 5 operated levels, 11 cases with 4 operated levels, 9 cases with 3 operated levels and 2 cases with 2 operated levels (Table 1). The average follow-up period of the patients was 20.14 ± 5.21 months.

**Table 1:** Summary of demographics and symptoms

Age	64.73 ± 10.31
Sex (n)	17 males, 9 females
Follow-up period (months)	20.14 ± 5.21
Five operated levels (n)	4
Four operated levels (n)	11
Three operated levels (n)	9
Two operated levels (n)	2
Operation time (min)	129.33 ± 15.74
Blood loss (ml)	356.13 ± 21.28
Preoperative VAS	8.04 ± 0.91
Postoperative VAS at 14th day of follow-up	1.79 ± 1.33
Postoperative VAS at final follow-up	1.62 ± 0.74
Preoperative ODI	61.33 ± 18.47
Postoperative ODI at 14th day of follow-up	43.26 ± 15.17
Postoperative ODI at final follow-up	39.24 ± 13.58

### Clinical evaluation

The clinical effects were evaluated by visual analogue scale (VAS) and Oswestry disability index (ODI) [10, 14]. In addition, the operated levels, the blood loss and operative time were also evaluated. We obtained and evaluated the VAS and ODI scores of patients before and immediately after surgery, 3 months, 6 months, 1 year after surgery, and at the end of follow-up period. In addition, the operated levels, the blood loss, operative time and adverse events were also recorded.

### Imaging examination

Imaging examinations were performed preoperatively and at the final follow-up postoperatively, including measurement of cobb angle, lumbar lordosis (LL), pelvic tilt (PT), apical vertebral translation (AVT), coronal vertical axis (CVA), sagittal vertical axis (SVA), sacral slope (SS) and pelvic incidence (PI). The radiological parameters were recorded before surgery and at the last follow-up after surgery. The cobb angle is formed by the intersection of two lines constructed from the superior and inferior vertebrae of the scoliosis curve. The LL is defined by the angle between the upper plane of the L1 lumbar vertebrae and the upper plane of the S1 sacral vertebrae. The PT is defined as the angle created by a line running from the sacral endplate midpoint to the center of the bifemoral heads and the vertical axis. The AVT is defined as the distance between the center of lumbar apical vertebra and central sacral vertical line (CSVL). The CVA is defined as the horizontal distance measured from a vertical plumb line centered in the middle of the C7 vertebral body to the CSVL. The SVA is measured as the distance between the C-7 plumb line and the posterior superior aspect of the S1 [15, 16]. The SS is defined as the angle between the sacral plate and the horizontal plane [17, 18]. The PI is defined as the angle between the line perpendicular to the sacral plate at its midpoint and the line connecting this point to the femoral head’s axis [18].

### Surgical management

The procedures of LSFCF were as follows: (1) after the success of anesthesia, the patients were placed in a prone position on the operating table. Take the operated level of L3–S1 for an example. Expose the L3–S1 facet joints and the lamina of the operated level bilaterally; (2) the L3–S1 pedicle screw fixation was performed bilaterally (Tianjin Zhengtian Medical Instrument Co., Ltd, China); (3) fenestration of the L3/4 level on the symptomatic side: decompression range should include medial to dorsal dural, lateral to L4 nerve root, cephalic to upper L4 nerve root sleeve, and caudal to L4 nerve root canal; (4) the interbody fusion was then performed following three principles: Firstly, if the symptomatic side of L3/4 is located on the concave side of the scoliosis, interbody fusion should be performed on the same side. In the meanwhile, on the asymptomatic side, decompression was not performed, and therefore the spinous process, interspinous ligament and lamina were preserved to maintain integrity of posterior spinal structures to the maximum; Secondly, if the symptomatic side of the segment is located on the convex side of the scoliosis, interbody fusion should be performed on the opposite side, that is, the concave side. Under this circumstance, fenestration on the opposite side should be performed; Thirdly, if the patient has sensory or motor impairment in both lower limbs, bilateral fenestration should be adopted, and interbody fusion should be performed on the concave side. The role of these principles is to ensure that interbody fusion is always performed on the concave side of the scoliosis. The cages used in this study were all manufactured by Johnson & Johnson (China) Medical Equipment Co., Ltd or Double Medical Technology Inc, China; (5) the L4/L5 and L5/S1 were managed in the same way; (6) the intraoperative fluoroscopy was used to confirm the good position of the screws; (7) special attention should be paid to the fact that convex compression and concave distraction were not performed after interbody fusion. All patients were immobilized in a waist support for 12 weeks postoperatively.

### Statistical analysis

Independent sample t-test was used to compare the mean value of VAS or ODI scores before and after surgery. The SPSS software for Windows (ver. 26.0; SPSS Inc, Chicago, IL, USA) was used to analyze the clinical data of patients. P < 0.05 was accepted as indicative of significant differences.

## Results

### Surgical outcome

The operation time and blood loss of patients underwent LSFCF were 129.33 ± 15.74 min and 356.13 ± 21.28 ml. The VAS and ODI scores significantly improved from 8.04 ± 0.91 preoperatively to 1.62 ± 0.74 postoperatively, and from 61.33 ± 18.47 preoperatively to 39.24 ± 13.58 postoperatively (*P* < 0.05). There was one patient with venous thrombosis in lower extremities (3.85%), pneumonia (3.85%) and urinary tract infection (3.85%) after surgery, respectively (Table 2). The overall complication rate was 11.54% and all patients were cured by conservative treatment before discharge. There was no case with cerebrospinal fluid leakage, cage subsidence, displacement, etc. during the follow-up period. No patients underwent secondary surgery during follow-up period and bony fusion was achieved in each patient at the final follow-up.

**Table 2:** Complications of the patients after surgery

	Cases (n = 26)
Deep tissue infection	0
Wound dehiscence	0
Thrombosis	1 (3.85%)
Pneumonia	1 (3.85%)
Cerebrospinal fluid leakage	0
Nerve root injury	0
Migration of prosthesis	0
Subsidence of prosthesis	0
Cardiopulmonary issues	0
Urinary tract infection or retention	1 (3.85%)
Ileus	0
Persistent radiculopathy	0
Acute renal failure	0
Reoperation rate	0
Total	11.54%

### Radiological examination

**Fig. 1:**
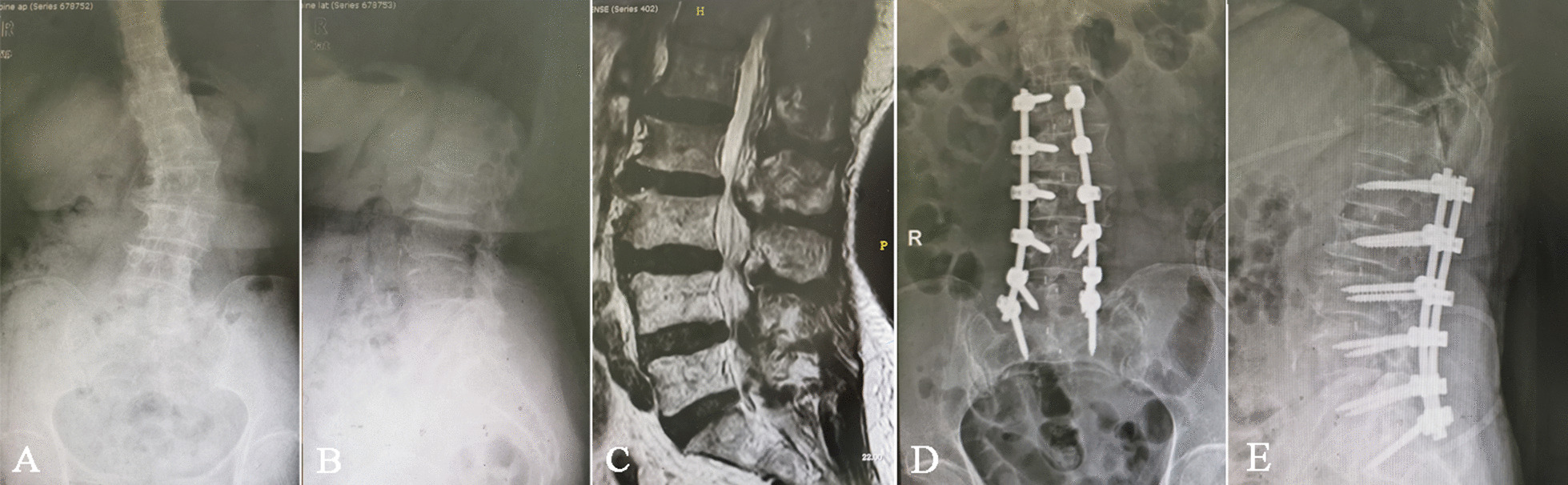
Preoperative lateral radiographic and MRI view (**A**–**E**) of a 72-year-old female with low back pain and numbness of the right lower limb for more than 3 years. Standing long cassette coronal and sagittal radiographs before surgery (**A**, **B**). The Cobb angle and lumbar lordosis were 26° and 42°, respectively. Standing long cassette coronal and sagittal radiographs at final follow-up after LSFCF surgery (**D**, **E**). The Cobb angle improved from 26° to 4°, and lumbar lordosis changed from 42° to 45°, respectively

**Fig. 2:**
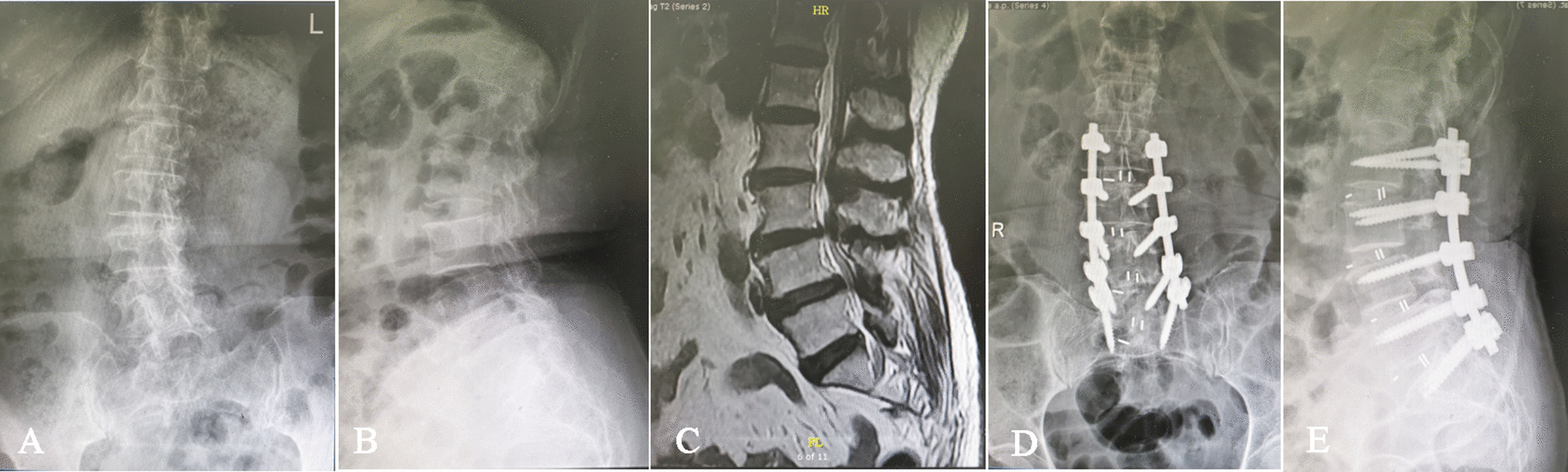
Preoperative lateral radiographic and MRI view (**A**–**E**) of a 75-year-old female with low back pain and intermittent claudication of both lower limbs for more than half a year. According to the preoperative standing long cassette coronal and sagittal radiographs, the Cobb angles and lumbar lordosis were 15° and 51°, respectively (**A**, **B**). The postoperative standing long cassette coronal and sagittal radiographs at final follow-up showed that the Cobb angle and lumbar lordosis improved from 15° to 6° and from 51° to 54°, respectively (**D**, **E**)

There was a significant improvement in cobb angles from 27.38 ± 5.24° preoperatively to 10.03 ± 3.13° at the final follow-up postoperatively (*P* < 0.05, Table 3). In the meanwhile, the LL significantly increased from 26.84 ± 4.38° preoperatively to 38.92 ± 4.46° at the final follow-up postoperatively (*P* < 0.05, Figs. 1, 2). Furthermore, the PT showed a significant decrease from 26.19 ± 3.09° preoperatively to 19.54 ± 2.35° at final follow-up postoperatively (*P* < 0.05). Finally, the AVT, CVA and SVA demonstrated significant improvements from 8.37 ± 2.41 mm to 3.83 ± 1.92 mm, from 27.18 ± 4.83 mm to 11.25 ± 2.74 mm, and from 32.87 ± 4.71 mm to 16.74 ± 3.22 mm at final follow-up postoperatively (*P* < 0.05). The SS exhibited an significant increase from 38.27 ± 7.37° to 44.73 ± 8.05° (*P* < 0.05), however, there is no significant change in the value of PI (*P* > 0.05).

**Table 3:** Radiological changes on imaging parameters of the patients with DLS after surgery

	Preoperative values	Postoperative values at 3rd after surgery	Postoperative values at final follow-up	*P* value
Cobb angles (°)	27.38 ± 5.24	9.94 ± 4.03	10.03 ± 3.13	*P* < 0.05
Lumbar lordosis (LL°)	26.84 ± 4.38	40.21 ± 4.84	38.92 ± 4.46	*P* < 0.05
Pelvic tilt (PT°)	26.19 ± 3.09	20.03 ± 3.17	19.54 ± 2.35	*P* < 0.05
Apical vertebral translation (AVT, mm)	8.37 ± 2.41	3.74 ± 2.05	3.83 ± 1.92	*P* < 0.05
Coronal vertical axis (CVA, mm)	27.18 ± 4.83	10.94 ± 3.01	11.25 ± 2.74	*P* < 0.05
Sagittal vertical axis (SVA, mm)	32.87 ± 4.71	16.11 ± 3.34	16.74 ± 3.22	*P* < 0.05
Sacral slope (SS°)	38.27 ± 7.37	45.19 ± 8.92	44.73 ± 8.05	*P* < 0.05
Pelvic incidence (PI°)	64.46 ± 8.79	63.97 ± 8.66	64.27 ± 8.45	*P* > 0.05

## Discussion

DS is mainly seen in elderly patients. It is often complicated with lumbar spinal stenosis, resulting in recurrent low back pain and intermittent claudication. The surgical treatment of DLS mainly includes decompression alone, short segment fixation and long segment fixation [10, 14, 19]. Decompression alone could relieve the symptoms of nerve elements compression, but it has no therapeutic effect on spinal instability and rotation of DLS. Furthermore, there is also the problem of accelerating the progression of spinal instability after operation [1]. Therefore, the long-term surgical effect of decompression alone is poor, and it is not an ideal method for the surgical treatment of DLS.

Long segment fixation can correct the coronal imbalance of scoliosis and reconstruct the stability of spine. However, long segment fixation has relatively higher rates of complications, including large amount of intraoperative bleeding, internal fixation displacement, loosening, nerve injury, etc. In addition, some studies have also indicated that even the correction effect of long segment fixation on sagittal imbalance of scoliosis is limited, and the reconstruction of physiological lordosis of lumbar spine is more important to alleviate the symptoms of low back pain than the correction of coronal imbalance for patients with DLS [10, 20].

Based on the problems of long segment fixation, short segment fixation is increasingly used in the surgical treatment of DLS patients. Short segment fixation can relieve the compression of nerve tissue through limited operated levels and can correct scoliosis deformity to a certain extent. Therefore, short segment fixation has the characteristics of simpler operation, shorter operation time and less complications compared with long segment fixation [21, 22]. However, studies have shown that short segment surgery is less effective than long segment fixation in correcting coronal deformity and reconstructing spinal stability of DLS [10, 23]. In view of previous problems, we modified the short segment surgery, namely LSFCF.

Compared with the traditional short segment fixation, the innovations of LSFCF mainly include the following three aspects: (1) we performed selective fenestration and decompression on the symptomatic side of DLS without total laminectomy, which is similar to the open-door laminoplasty for cervical spondylotic myelopathy. Therefore, LSFCF can shorten the operation time and reduce the incidence of cerebrospinal fluid leakage; (2) We adopted the concave side of DLS for interbody fusion, which is more conducive to obtain ideal correction of coronal imbalance; (3) For cases with straight lumbar curvature or even kyphosis, multi-segment interbody fusion can better restore the physiological lordosis of lumbar spine.

At the symptomatic convex side, we only performed fenestration with preservation of facet joints. Therefore, it does not affect the spinal stability. On the other hand, the purpose of interbody fusion on the asymptomatic concave side is to better correct the coronal imbalance and increase the physiological curvature of lumbar lordosis simultaneously. During LSFCF, we do not advocate the convex compression, which will reduce the height of intervertebral foramen and could cause neurological symptoms. For these reasons, we believe that LSFCF does not significantly increase surgical trauma.

Previous studies have demonstrated that the use of lordotic cages in lumbar interbody fusion resulted in a significant increase in lordosis at operative levels [24–26]. The results of the current study showed that there were significant increases of LL in patients with DLS after LSFCF (*P* < 0.05). Therefore, it can be concluded that through the interbody fusion of multiple degenerative segments, LSFCF is conducive to restore the lordosis and correct the sagittal imbalance of the degenerative lumbar spine. The fusion range of LSFCF should follow the following two principles: (1) the fixation range must span the apical vertebra of lumbar scoliosis; (2) interbody fusion should be performed for each compression level at concave side of DLS.

Furthermore, the coronal imbalance in terms of cobb angles, AVT, CVA and SVA has been significantly improved after LSFCF at the final follow-up (*P* < 0.05). LSFCF through the concave approach for lumbar interbody fusion can enhance the distraction effect at the concave side. The anterior and middle columns of the lumbar spine can be distracted by selecting the cage with slightly higher height than the intervertebral space, which can better correct the coronal deformity of the lumbar spine than using posterior inter-screws distraction alone.

Patients with DLS are usually complicated with LSS. In addition to correcting spinal deformity, relieving the compression of nerve tissue is also the key to the success of surgery. The LSFCF surgery preserves the lumbar posterior column structure on the convex side to the greatest extent. Through fenestration on the concave side and intervertebral distraction, it can effectively relieve the neural elements compression. The ODI scores of all cases were significantly improved at the last follow-up after operation, which confirmed the effectiveness of LSFCF. In addition, due to the simplification of operation procedures, the rate of nerve tissue injury is reduced and there was no case with cerebrospinal fluid leakage or nerve root injury in our series.

Lenke–Silva classification have been widely used in the surgical management of DLS [27–29]. The patients enrolled in this study included some cases with straight lumbar curvature or even kyphosis. However, these cases did not show global spinal imbalance. Therefore, the DLS patients with Lenke–Silva grade V and VI were not included in our study, and these patients are not suitable for LSFCF surgery. In fact, LSFCF can be applied to patients with Cobb angle less than 45° and no global spinal imbalance. For DLS patients with Lenke–Silver grade I–IV, promising clinical effects can be achieved by LSFCF. However, long segment fixation should be the preferred surgical treatment for DLS patients with Lenke–Silver grade V–VI.

There are still disadvantages in LSFCF. Firstly, the operation space at concave side is relatively small, and the incidence of nerve root injury is likely to increase during fusion operation. Therefore, we suggest that the transforaminal approach should be adopted at concave side to ensure sufficient space to insert the cage to address this issue. Secondly, the intervertebral space at the concave side is usually relatively narrow, and it is more difficult to implant the cage than that at the convex side. Our solution is to make a small incision in the annulus fibrosus and then slowly insert it with a curved probe to explore the accurate direction of the intervertebral space. This strategy can be very effective to ensure the accurate placement of the cage, and cage subsidence and nerve injury did not occur at each fusion level in our series.

The limitations of this study mainly include the following two aspects: Firstly, the follow-up time of the study is relatively short. Secondly, there is a lack of control group. In the future, we will conduct long-term clinical control studies to further investigate the effectiveness and safety of LSFCF.

## Conclusion

For patients with DLS combined with LSS, LSFCF surgery can significantly improve their low back pain and neurological dysfunction, and effectively reduce the incidence of surgical trauma and complications. It is an effective method for the treatment of DLS combined with LSS.

## Data Availability

The data that support the findings of this study are available on request from the corresponding author. The data that can be provided will be provided in a de-identified manner.

## Abbreviations

LSFCF: Lumbar selective fenestration and concave-side fusion
DLS: Degenerative lumbar scoliosis
LSS: Lumbar spinal stenosis
VAS: Visual analogue scale
ODI: Oswestry disability index
